# From organelles to therapy: rethinking combined hepatocellular-cholangiocarcinoma

**DOI:** 10.3389/fcell.2026.1787784

**Published:** 2026-04-15

**Authors:** Tiezhong Zhang, Kangshuai Li, Qi Li, Qiang Gao, Lixin Du, Jian Deng, Zhuohan Cao, Sen Guo, Zongli Zhang

**Affiliations:** Department of General Surgery, Qilu Hospital, Cheeloo College of Medicine, Shandong University, Jinan, Shandong, China

**Keywords:** cholangiocarcinoma, hepatocellular carcinoma, histopathological characteristics, multidisciplinary treatment, primary liver cancer, review

## Abstract

Combined hepatocellular-cholangiocarcinoma (cHCC-CCA) is a rare primary malignant hepatic neoplasm, defined by the concurrent presence of hepatocellular carcinoma (HCC) and cholangiocarcinoma (CCA) components, which vary in proportion and degree of differentiation. Characterized by insidious onset, high invasiveness, and marked heterogeneity, cHCC-CCA often eludes early diagnosis, leading to a generally dismal prognosis. Its survival outcomes typically fall between those of HCC and intrahepatic cholangiocarcinoma (iCCA). Epidemiological data derived from surgical resection specimens and percutaneous biopsy samples indicate that cHCC-CCA accounts for approximately 0.4%–14.2% of all primary liver cancers. Due to its rarity, standardized treatment protocols are currently lacking. Surgical resection and liver transplantation are considered the primary potential curative approaches. However, only a minority of patients meet surgical criteria at diagnosis, and postoperative recurrence rates are substantially high. For non-surgical candidates, local or systemic therapies are generally administered based on treatment regimens for HCC or iCCA. Additionally, the pronounced genetic and molecular heterogeneity of cHCC-CCA significantly compromises the efficacy of current therapeutic strategies. Its unique biological behaviors, histological features, and immunophenotypic profiles present multifaceted challenges to diagnosis, treatment, and research endeavors. This review aims to comprehensively synthesize the classification systems and pathological characteristics of cHCC-CCA, with a particular focus on the underlying organelle dysfunction. By integrating advances in clinical diagnosis and management, we seek to enhance disease awareness and provide a new reference for clinical practice.

## 1 Introduction

Combined hepatocellular-cholangiocarcinoma (cHCC-CCA) is a relatively rare subtype of primary liver cancer that exhibits features of both hepatocytic and biliary differentiation (Tang et al., 2025; Komuta and Yeh, 2020). Current clinical epidemiological studies indicate that cHCC-CCA accounts for 0.4%–14.2% of all primary liver cancers (Ilyas et al., 2023; Wang H. et al., 2025; Ma L. et al., 2025; Zou et al., 2025). The wide reported incidence range reflects substantial heterogeneity across studies in terms of study populations, specimen types, and diagnostic criteria. First, the study population significantly influences incidence estimates. In clinical practice, due to the lack of a biopsy or sampling bias in needle biopsies, many patients who do not undergo surgical resection are frequently misdiagnosed as hepatocellular carcinoma (HCC) or intrahepatic cholangiocarcinoma (iCCA). Second, geographic variation is evident, with Asian series generally reporting higher proportions than Western series. This suggests that differences in the underlying etiology of liver disease may play a contributing role. Specifically, hepatitis B is more predominant in Asia, while hepatitis C and metabolic disease are more common in the West. Third, and most importantly, evolving diagnostic criteria across WHO classifications have substantially affected reported frequencies. Studies applying the 2010 WHO classification, which included “subtypes with stem-cell features,” reported higher rates than those using the 2019 WHO classification, eliminating this subcategory and re-centering diagnosis on the definitive coexistence of both lineages. Finally, selection bias in single-center retrospective series further contribute to the wide range. Although it is widely accepted in the academic community that surgical treatment aimed at radical cure should be the first choice for resectable lesions, most patients are diagnosed at a stage where surgery is unfeasible, and postoperative recurrence rates remain high (Ye et al., 2024; Sun et al., 2025). This article systematically reviews the clinical classification, diagnostic criteria, molecular biology, and treatment strategies for cHCC-CCA, with the aim of enhancing the understanding of this unique tumor and providing a reference for clinical management.

## 2 Cellular origin and phenotypic plasticity of cHCC-CCA

### 2.1 Cellular phenotype and histogenesis of cHCC-CCA

**FIGURE 1:**
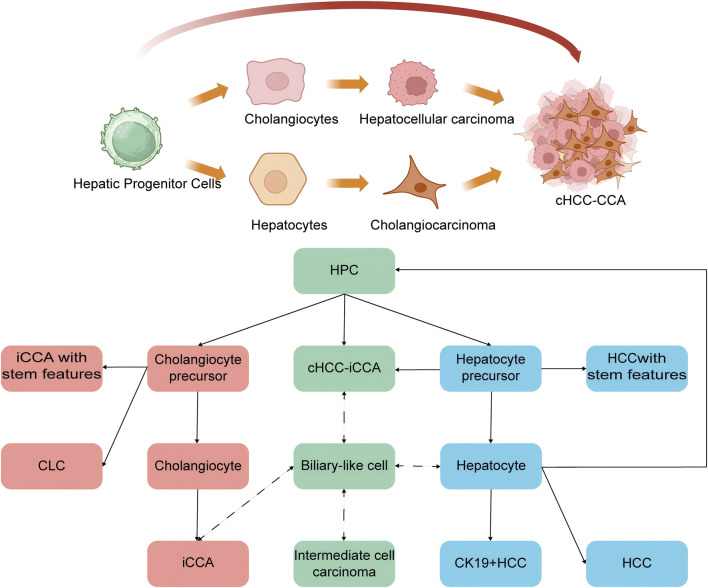
Cellular origin and histological phenotype of cHCC-CCA Two main theories explain cHCC-CCA histogenesis: transdifferentiation (committed hepatocytes or cholangiocytes switch lineage) and progenitor cell origin (bipotent hepatic progenitor cells give rise to both components). These mechanisms may coexist, contributing to tumor heterogeneity.

The cellular origin of cHCC-CCA remains controversial, with two primary hypotheses prevailing: the “transdifferentiation theory” and the “progenitor cell origin theory” (Calderaro et al., 2023; Liu et al., 2021). The transdifferentiation theory posits that cHCC-CCA represents not a discrete clinicopathological entity, but a transitional phenotype wherein HCC and iCCA components demonstrate bidirectional lineage plasticity (Komuta and Yeh, 2020; Calderaro et al., 2023). This theory proposes a clonal evolution from either committed malignant hepatocytes or cholangiocytes, which subsequently undergo phenotypic reprogramming to express markers of the alternate lineage during tumor progression (Kim et al., 2019). Notably, the postulated predominant direction of this transdifferentiation exhibits geographical divergence in scholarly perspective. In Asia, the observation of shared clinicopathological features with HCC has led to the proposition that cHCC-CCA predominantly arises from HCC transdifferentiating towards an iCCA phenotype (Kim et al., 2019; P et al., 2023; Xue et al., 2019; Ohni et al., 2024; Muhammad et al., 2024). Conversely, Western molecular profiling studies identifying common genetic drivers between cHCC-CCA and iCCA support an alternative view, suggesting an origin from iCCA acquiring hepatocytic differentiation (Zhao et al., 2022; Coulouarn et al., 2012). In contrast, the progenitor cell origin theory proposes that cHCC-CCA originates directly from bipotent hepatic progenitor cells (HPCs) (Rosenberg et al., 2022; Choi et al., 2021; Zhao et al., 2016). This theory holds that malignant transformation of HPCs drives divergent differentiation along both hepatocytic and biliary lineages concurrently, thereby generating the definitive mixed phenotype. This hypothesis has accrued support from recent experimental validation. For instance, lineage-tracing studies by Rosenberg et al. demonstrated that forkhead box protein L1 (Foxl1) + progenitor cells residing in the canals of Hering are the specific cells of origin for cHCC-CCA in murine models of chronic liver injury (Xu et al., 2024). In summary, the histogenesis of cHCC-CCA is characterized by significant heterogeneity. The theories outlined above are not mutually exclusive and may operate in concert to drive tumorigenesis, offering a plausible explanation for the tumor’s complex histology and diverse clinical behavior (Calderaro et al., 2023) (Figure 1).

### 2.2 Molecular genetic characteristics of cHCC-CCA

Molecular genetic studies of cHCC-CCA have not only unveiled its complex features, positioning it between HCC and iCCA, but also provided insights into its tumor heterogeneity. Research by Joseph et al. found that genetic alterations in cHCC-CCA, such as telomerase reverse transcriptase (TERT) promoter mutations (80%) and tumor protein 53 (TP53) mutations (80%), are highly similar to those in HCC, highlighting a genetic kinship (Joseph et al., 2019). TERT promoter and TP53 are among the most frequently mutated genes in cHCC-CCA, which are considered potential initiating events in tumorigenesis (Eschrich et al., 2023; Zhang et al., 2024). Notably, isocitrate dehydrogenase (IDH) 1/2 mutations have also been observed in HCCs with cholangiocytic features, suggesting their potential role in driving the tumor toward a biliary phenotype (Eschrich et al., 2023; Cancer Genome Atlas Research NetworkCancer Genome Atlas Research Network, 2017). These molecular genetic findings corroborate the aforementioned view of the highly heterogeneous cellular origin of cHCC-CCA. Conversely, other scholars argue that cHCC-CCA is genetically closer to iCCA, whole-genome and transcriptome analyses further support that cHCC-CCA exhibits a downregulation of hepatocellular differentiation programs alongside a shift toward biliary lineage differentiation, given the high frequency of chromosomal instability and loss of heterozygosity on chromosomes 3p and 14q, which occur in >50% of both cHCC-CCA and iCCA cases but in <10% of HCC cases (Zhao et al., 2022; Gurzu et al., 2024; Rossner et al., 2023).

And, inhibition of the nuclear factor kappa B (NF-κB) signaling pathway has been shown to drive a phenotypic shift from MYC-driven HCC to cHCC-CCA, suggesting that specific genetic and signaling alterations can directly steer tumor evolution (Schaub et al., 2018). Furthermore, through a systematic analysis of 133 cHCC-CCA cases alongside control samples of HCC and iCCA, Xue et al. found that separate cHCC-iCCA cases could arise from either monoclonal or multicellular origins, whereas the mixed and combined types of cHCC-iCCA were exclusively monoclona (Xue et al., 2019). Among these two monoclonal subtypes, molecular analysis revealed that the combined subtype exhibited features more closely resembling iCCA, while the mixed subtype displayed HCC-like characteristics (Xue et al., 2019; Azizi et al., 2020). All of this proves that cHCC-CCA is not a homogeneous entity but rather comprises subtypes with distinct cellular origins and molecular characteristics at the molecular level (Azizi et al., 2020).

In addition, several other developmentally regulated signaling pathways have been implicated in cHCC-CCA pathogenesis, though their roles remain less well-defined. In cHCC-CCA, nuclear accumulation of beta-catenin (β-catenin) has been observed in a subset of cases, particularly those with progenitor cell features, suggesting that the wingless-related integration site (Wnt) pathway plays a potential role in maintaining stemness and enabling bidirectional differentiation (Coulouarn et al., 2012). Notch signaling, a master regulator of biliary specification during development, promotes cholangiocyte differentiation when activated in hepatocytes (Ma W. et al., 2025; Yang et al., 2025). In cHCC-CCA, Notch pathway activation may drive the biliary component by suppressing hepatocyte fate and inducing cholangiocytic gene programs (Ma W. et al., 2025; Yang et al., 2025). Hedgehog signaling, which mediates epithelial-mesenchymal interactions during liver development, has been shown to promote desmoplasia and invasion in iCCA and may contribute to the stromal-rich phenotype observed in the iCCA component of cHCC-CCA(32). Protein Kinase B (AKT)/mammalian Target of Rapamycin (mTOR) signaling, a central regulator of cell growth and metabolism, is frequently activated in both HCC and iCCA and has been associated with aggressive behavior (Jeng et al., 2024; Ferrin et al., 2020; Xue et al., 2024; Liang et al., 2024; Falcomata et al., 2021). Based on evidence from HCC and iCCA, it is reasonable to infer that in cHCC-CCA, AKT activation may support the metabolic demands of dual-lineage maintenance by promoting aerobic glycolysis and suppressing apoptosis. Neuroblastoma RAS viral oncogene homolog (N-RAS) mutations, though rare in HCC, have been identified in a subset of iCCAs and may contribute to the biliary phenotype in cHCC-CCA through mitogen-activated protein kinase (MAPK) pathway activation (Rosenberg et al., 2022; Jeng et al., 2023; Gruttadauria et al., 2021). Due to the low incidence and detection rate of cHCC-CCA and its highly heterogeneous molecular profile, the current understanding of its genetic characteristics remains incomplete (Ilyas et al., 2023; Ye et al., 2024). A deeper dissection of its genetic landscape will help elucidate the etiological nature, mechanisms of development, therapeutic responses, and prognosis of cHCC-CCA, while also providing clues for identifying new therapeutic targets (Choi and Ro, 2022). Future research should focus on validating these findings in larger cohorts and exploring the translation of these molecular features into effective targeted and immunotherapeutic strategies.

## 3 Dysfunction of cellular organelles in the pathogenesis of cHCC-CCA

Cellular organelles, as the core functional units of cellular life activities, serve as the material basis for malignant tumor behavior when their homeostasis and function are disrupted (Gruttadauria et al., 2021; Choi and Ro, 2022). Although direct research focusing on organelles in cHCC-CCA is scarce, extensive studies on HCC and iCCA provide critical clues and a rational basis for inferring potential organelle dysregulation in cHCC-CCA(40, 41).

### 3.1 Direct evidence in cHCC-CCA: ultrastructural observations

Direct evidence of organelle dysfunction in cHCC-CCA is scarce, but recent advances in establishing cHCC-CCA cell lines have provided the morphological insights. The recently characterized cHCC-CCA cell line CHC-X1, derived from a patient with combined phenotype, has been examined by transmission electron microscopy. Key observations include: mitochondrial abnormalities, abundant rough endoplasmic reticulum (RER), increased lysosomal abundance.

The cytoplasm contains numerous irregularly shaped, swollen mitochondria with disrupted cristae. This morphology is suggestive of mitochondrial stress, potentially reflecting altered metabolic demands associated with maintaining dual lineage identity. The cytoplasm of CHC-X1 cells contained extensive networks of RER, indicating heightened protein synthesis and folding activity. While these initial findings establish organelle abnormalities as a morphological hallmark of cHCC-CCA, the mechanistic consequences of these features remain critical, unexplored questions.

### 3.2 Insights from HCC and iCCA: a hypothesis-generating framework

While direct evidence in cHCC-CCA is limited, the biological overlap between cHCC-CCA and its pure counterparts provides a rational basis for inferring organelle-level mechanisms. Here, we synthesize findings from HCC and iCCA not as established facts in cHCC-CCA, but as a framework to generate testable hypotheses regarding organelle dysfunction in this biphenotypic tumor (Liu et al., 2025).

#### 3.2.1 Mitochondria: metabolic flexibility and lineage plasticity

Mitochondria are central to cellular metabolism, redox balance, and apoptosis. In iCCA, the chemotherapeutic agent cisplatin has been shown to degrade inverted formin 2 (INF2), a protein located at ER mitochondria contact sites (Ding et al., 2024). This degradation occurs through two parallel pathways: activation of the ubiquitin-proteasome system and induction of ER-phagy, resulting in suppressed mitochondrial fission and excessive mitochondrial fusion (Reggiori and Molinari, 2022; Jin et al., 2024; Chen et al., 2025). The resulting shift toward a hyperfused mitochondrial network promotes survival under cisplatin-induced stress.

In HCC, the mitochondrial carrier protein solute carrier family 25 member 39 (SLC25A39) has been identified as a key oncogenic factor that regulates intramitochondrial reactive oxygen species (ROS) levels and cytochrome c release, thereby driving tumor progression (Yuan et al., 2025; Lv et al., 2025). Targeting mitochondrial metabolism has therefore emerged as a potential therapeutic strategy in HCC. Mitochondrial dynamics are also closely linked to tumor behavior. For instance, inhibition of HSP90 in HCC cells triggers a compensatory increase in mitochondria-derived vesicles (MDVs), small double-membraned vesicles that bud from mitochondria and carry selected mitochondrial cargo (including oxidized proteins and mitochondrial DNA fragments) to lysosomes for degradation (Liu et al., 2025). This process represents a quality control mechanism that removes damaged mitochondrial components without engaging whole-organelle mitophagy. However, in the context of heat shock protein 90 (HSP90) inhibition, this compensatory MDV response becomes hijacked for pro-metastatic purposes (Liu et al., 2025; Tan et al., 2022). The MDVs are subsequently packaged into extracellular vesicles (EVs) and released into the tumor microenvironment, where they are taken up by neighboring cancer cells and stromal cells (Sager et al., 2022; Kumar, 2024). Once internalized, these MDV-containing EVs transfer mitochondrial contents that can reprogram recipient cell metabolism through the introduction of mitochondrial components, activate stress signaling pathways such as ROS-mediated signaling to promote invasive phenotypes, and prime the pre-metastatic niche by educating stromal cells and suppressing local immune surveillance (Tan et al., 2022; Mishra and Deep, 2024). This intercellular transfer of mitochondrial material *via* the MDV-EV axis thus creates a mechanism by which stressed cancer cells can “communicate” their adaptive state to the broader tumor ecosystem, collectively enhancing metastatic capacity (Liu et al., 2025).

The swollen, irregular mitochondria observed in CHC-X1 cells may reflect a state of mitochondrial stress. We suggest that the balance between mitochondrial fission and fusion may determine whether cHCC-CCA cells adopt a more HCC-like (fission-dominant, glycolytic) or iCCA-like (fusion-dominant, oxidative) metabolic phenotype (Tang et al., 2025). If SLC25A39-mediated ROS regulation operates similarly in cHCC-CCA, it could represent a therapeutic vulnerability. These findings lead us to propose the testable hypothesis that pharmacological inhibition of SLC25A39 or modulation of mitochondrial dynamics such as dynamin-1-like protein (DRP1) inhibitors or mitochondrial division inhibitor-1 (Mdivi-1) may selectively suppress one lineage component while sparing the other, potentially revealing lineage-specific dependencies (Liu et al., 2025; Jin et al., 2024; Tan et al., 2022).

#### 3.2.2 Endoplasmic reticulum: protein homeostasis and differentiation

The ER is the primary site for synthesis, folding, and modification of secretory and membrane proteins. ER homeostasis disruption is strongly linked to tumor progression. Central to ER quality control is the suppressor of Lin-12-like protein 1 - HMG-CoA reductase degradation one homolog (SEL1L-HRD1) complex, a key mediator of ER-associated degradation (ERAD) (Lin et al., 2024; Bhattacharya et al., 2022). This complex functions as a surveillance system that recognize misfolded or unassembled proteins in the ER lumen, retrotranslocates them across the ER membrane, and targets them for ubiquitin-proteasome degradation in the cytosol. By clearing potentially toxic protein aggregates and preventing ER stress, ERAD maintains cellular proteostasis and supports the high secretory demands of cancer cells (Bhattacharya et al., 2022).

In HCC, inhibition of the SEL1L-HRD1 complex suppresses cancer cell proliferation and migration, revealing that ERAD activity actively supports tumor growth. Mechanistically, SEL1L-HRD1-mediated ERAD promotes HCC progression through several interconnected pathways (Chen et al., 2025; Bhattacharya et al., 2022; Guan et al., 2020). First, it enables cancer cells to tolerate oncogene-driven protein synthesis stress by efficiently degrading misfolded proteins that would otherwise trigger apoptotic unfolded protein response (UPR) signaling. Second, it selectively degrades tumor suppressor proteins and pro-apoptotic factors, tipping the balance toward cell survival. Third, it supports the secretion of pro-tumorigenic factors such as growth factors, cytokines, and extracellular matrix remodeling enzymes, which require proper folding in the ER before secretion (Wang et al., 2023). Thus, the SEL1L-HRD1 ERAD pathway represents a dependency in HCC cells that cope with chronic proteotoxic stress, and its inhibition disrupts this adaptive mechanism, leading to growth suppression (Wang et al., 2023).

The abundant RER observed in CHC-X1 cells suggests that cHCC-CCA cells may be under chronic ER stress, potentially activating the UPR. In other systems, UPR activation can drive cellular differentiation and lineage commitment. We suggest that ER stress-induced UPR signaling may promote biliary differentiation *via* downstream effectors such as TGF-β, which has been implicated in cHCC-CCA pathogenesis. The ER-mitochondria lipid synthesis axis may be particularly active in cHCC-CCA, where dual-lineage maintenance imposes high demands on both mitochondrial and secretory pathway function. A testable prediction is that Inhibition of ER-mitochondria lipid transfer, such as *via* targeting phosphate cytidylyltransferase2 (PCYT2) or ER-mitochondria contact sites, may disrupt the metabolic coordination required for biphenotypic persistence, selectively impairing cHCC-CCA cell viability.

#### 3.2.3 Lysosomes and autophagy: stress adaptation and therapy resistance

**FIGURE 2:**
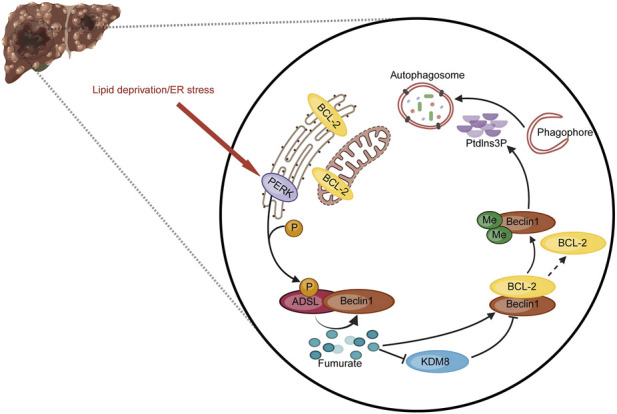
Schematic diagram of the ADSL–fumarate–KDM8–Beclin1 axis regulating autophagy under lipid deprivation and ER stress in liver cancer. Under lipid deprivation or ER stress, PERK phosphorylates ADSL, enhancing its binding to Beclin1. ADSL-generated fumarate inhibits KDM8, leading to Beclin1 dimethylation, which releases Beclin1 from BCL2 inhibition and activates autophagy, enabling liver cancer cells to survive metabolic stress. Blunt arrows indicate inhibition; solid arrows represent molecular transformation or catalysis; dashed arrows denote dissociation.

Lysosome-mediated autophagy plays a dual role in cancer. It can suppress tumorigenesis by eliminating damaged organelles and proteins, but also sustain tumor cell survival under nutrient-deprived conditions (Wang et al., 2025b; Mohammed et al., 2024; Kar et al., 2022). In HCC, a recently elucidated mechanism reveals how metabolic reprogramming directly regulates autophagy initiation. The purine synthesis enzyme adenylosuccinate lyase (ADSL) is phosphorylated at serine 140 by protein kinase R-like endoplasmic reticulum kinase (PERK) in response to lipid deprivation or ER stress. This phosphorylation enhances the association between ADSL and the core autophagy regulator Bcl-2 interacting coiled-coil protein 1 (Beclin1) (Wang et al., 2025b; Wang et al., 2025c). ADSL produces fumarate as a byproduct of its enzymatic activity, and the Beclin1-associated ADSL generates locally elevated fumarate concentrations. Fumarate inhibits the activity of lysine demethylase 8 (KDM8), a lysine demethylase associated with the ADSL-Beclin1 complex, leading to accumulation of Beclin1 K117 dimethylation. This specific methylation modification disrupts the inhibitory interaction between Beclin1 and BCL2, releasing Beclin1 to participate in the class III phosphatidylinositol 3-kinase (class III PI3K) complex that nucleates autophagosome formation. Thus, through this ADSL-fumarate-KDM8-Beclin1 axis, metabolic stress signals are directly transduced to the core autophagy machinery, enabling HCC cells to adapt to nutrient-deprived microenvironments and promoting tumor growth (Wang et al., 2025c) (Figure 2). In iCCA, particularly in subtypes with specific mutations such as kirsten rat sarcoma viral oncogene homolog (KRAS), lysosomes enable metabolic adaptation through macropinocytosis, a process by which tumor cells engulf extracellular fluid containing macromolecules (e.g., proteins), which are then degraded in lysosomes to generate amino acids and lipids for proliferation. Inhibitors targeting this pathway can induce metabolic crisis and cell death by severing the “nutrient supply chain” of cancer cells (Lee et al., 2024; Peixoto et al., 2025).

The increased lysosomal abundance in cHCC-CCA cells raises critical questions: Does this represent enhanced autophagic flux, and if so, does it support survival under metabolic stress or actively maintain the biphenotypic state? Alternatively, could it reflect lysosomal dysfunction, leading to impaired clearance of damaged organelles and accumulation of autophagic substrates? If cHCC-CCA cells depend on macropinocytosis, a possibility suggested by the presence of an iCCA component, they may be vulnerable to lysosome-targeting agents such as chloroquine or hydroxychloroquine. This leads to the testable prediction that cHCC-CCA cell lines with prominent iCCA features will demonstrate greater sensitivity to lysosomal inhibition compared to those with predominantly HCC-like characteristics.

#### 3.2.4 Primary cilia: a cholangiocyte-specific organelle

The primary cilium is a microtubule-based organelle that functions as a cellular antenna, sensing extracellular signals and coordinating cellular responses. In cholangiocytes, primary cilia are particularly abundant and serve as core platforms for coordinating DNA damage repair. Loss of primary cilia contributes to genomic instability and has been implicated in cholangiocarcinogenesis (Peixoto et al., 2025).

The iCCA component of cHCC-CCA may arise from cells that have lost primary cilia function, leading to accumulated DNA damage and malignant transformation. A key, testable prediction arising from this reasoning is that cHCC-CCA tumors with a predominant iCCA component will show reduced primary cilia frequency or disrupted ciliary signaling compared to HCC-predominant tumors.

#### 3.2.5 Autophagosome: a functional organelle

The autophagosome represents a pivotal structure within the autophagic pathway, defined as a double-membraned vesicular organelle (Fujioka and Noda, 2025). It engulfs cytoplasmic cargo, such as damaged organelles and misfolded proteins, and transports them to lysosomes for degradation. Traditionally perceived as a transient intermediate within the autophagic flux, its principal function has been ascribed to the degradation and recycling of cellular components (Xia et al., 2021). However, accumulating evidence has redefined the autophagosome as a distinct and multifunctional organelle whose biological significance extends substantially beyond this conventional role. In the context of tumor biology, the autophagosome assumes multifaceted functions: it not only directly modulates cell survival through its biogenesis and abundance, but also serves as a signaling platform and cargo carrier implicated in critical processes such as metastasis, immune modulation, and metabolic adaptation (Xia et al., 2021; Li et al., 2025).

While the preceding discussion has focused on autophagy as a process, the autophagosome itself, the double-membraned vesicle that engulfs cytoplasmic cargo, is increasingly recognized as a functional organelle with roles beyond simply delivering cargo to lysosomes. Although direct evidence in cHCC-CCA is absent, insights from HCC and CCA suggest that autophagosomes may contribute to tumor biology through at least three distinct mechanisms, each generating testable hypotheses for cHCC-CCA(64).

In HCC, the number of autophagosomes, marked by microtubule-associated protein one light chain 3 (LC3), is significantly higher in metastatic lesions than in paired primary tumors (Li et al., 2025; Liu et al., 2026). Using a pulmonary metastasis mouse model, researchers demonstrated that autophagosome formation is specifically activated during the metastatic colonization phase when disseminated tumor cells adapt to a new microenvironment, but not during cell migration, invasion, or detachment from the extracellular matrix (Li et al., 2025; Yi et al., 2015). This suggests that autophagosomes, as structural containers, facilitate the survival and outgrowth of metastasizing HCC cells in foreign tissues.

Beyond their intracellular degradative function, autophagosomes can also be released from tumor cells and act as extracellular vesicles. In HCC, autophagosomes isolated from tumor cells have been successfully used as a vaccine to load dendritic cells, enhancing cross-presentation of tumor antigens and stimulating a cytotoxic T-cell response (Liu et al., 2026). This indicates that autophagosomes are carriers of immunogenic material and can modulate the anti-tumor immune response. In cHCC-CCA, it is unknown whether autophagosomes from the HCC and iCCA components carry distinct antigen repertoires, and whether their release shapes the unique tumor microenvironment of this biphenotypic cancer.

The formation of an autophagosome requires a massive and rapid supply of membrane lipids. Emerging evidence points to a bidirectional relationship between autophagosomes and lipid droplets (LDs). LDs not only provide lipids for autophagosome membrane synthesis, but are themselves selectively degraded by autophagy or lipophagy to fuel mitochondrial beta-oxidation (Liu et al., 2026; Petan et al., 2018). This lipid-mediated crosstalk supports cancer cell survival under metabolic stress. The “abundant RER” observed in CHC-X1 cells may be the source of these membranes. For cHCC-CCA, the dual metabolic demands of maintaining both hepatocytic and cholangiocytic lineages may create a heightened dependence on this LD-autophagosome-mitochondria axis for metabolic flexibility, a vulnerability that could be therapeutically exploited.

In summary, the autophagosome is not merely a transient intermediate but a dynamic organelle whose abundance, cargo, and membrane dynamics directly influence metastasis, immune recognition, and metabolic adaptation. The ultrastructural observation of increased lysosomes in CHC-X1 cells hints at an active autophagic pathway, but the key question remains: Are autophagosomes themselves functionally engaged in these processes in cHCC-CCA, and if so, does their role differ between the HCC-like and iCCA-like components? Answering this will require direct quantification of autophagosome formation in cHCC-CCA models under conditions of stress, therapy, and immune surveillance.

### 3.3 A working model: organelle dysfunction in cHCC-CCA pathogenesis

Based on the direct observations in cHCC-CCA and extrapolations from HCC and iCCA, we propose a preliminary working model linking organelle dysfunction to the unique features of cHCC-CCA (Table 1).

**TABLE 1:** Testable hypotheses arising from the organelle dysfunction model in cHCC-CCA.

Hypothesis	Experimental approach	Predicted outcome
H1: Mitochondrial fission/fusion balance determines lineage preference	Treat cHCC-CCA cell lines with DRP1 inhibitor (Mdivi-1) or MFN2 agonist; assess lineage marker expression (HepPar-1, AFP vs. CK7, CK19) *via* qPCR and IHC	DRP1 inhibition shifts cells toward iCCA-like phenotype (↑CK7/CK19, ↓HepPar-1); MFN2 activation promotes HCC-like phenotype
H2: ER stress drives biliary differentiation *via* UPR-TGF-β crosstalk	Treat cHCC-CCA cells with ER stress inducers (tunicamycin, thapsigargin) ± TGF-β receptor inhibitor (SB431542); assess CK7/CK19 expression and UPR markers (XBP1s, ATF4, CHOP)	Time-dependent increase in biliary marker expression; effect attenuated by TGF-β inhibition
H3: Lysosomal activity supports survival under metabolic stress in iCCA-predominant tumors	Culture cHCC-CCA cell lines (stratified by lineage predominance) in nutrient-limited medium ± chloroquine or hydroxychloroquine; assess viability, apoptosis, and autophagic flux (LC3-II/LC3-I ratio, p62 levels)	iCCA-predominant lines show greater sensitivity to lysosomal inhibition under stress; HCC-predominant lines relatively resistant
H4: Combined organelle targeting produces synergistic anti-tumor effects	Treat cHCC-CCA xenografts with: (A) DRP1 inhibitor alone, (B) chloroquine alone, (C) combination; assess tumor growth, lineage marker expression, and apoptosis (TUNEL)	Combination therapy shows greater tumor inhibition than either agent alone; reduced expression of both lineage markers
H5: Primary cilia loss correlates with iCCA predominance and genomic instability	Perform immunofluorescence for acetylated α-tubulin (cilia marker) and γ-H2AX (DNA damage marker) in cHCC-CCA tissue microarrays; correlate with CK7/CK19 and HepPar-1 expression	iCCA-rich regions show reduced cilia frequency and increased γ-H2AX foci compared to HCC-rich regions
H6: Autophagosome formation is required for metastatic colonization of iCCA component	Generate cHCC-CCA cell lines with ATG5 or ATG7 knockdown; inject into tail vein metastasis model; quantify lung metastatic burden and lineage composition	ATG5/7 knockdown reduces metastatic outgrowth, particularly affecting CK7+/CK19+ metastatic deposits
H7: Autophagy-related protein expression (LC3) predicts postoperative prognosis	Perform LC3 IHC on resected cHCC-CCA tissue microarray; correlate expression levels with clinicopathological features (vascular invasion, LN metastasis, stage) and survival outcomes	High LC3 expression independently predicts improved OS and DFS, consistent with prior single-center study

All hypotheses are proposed based on extrapolations from HCC and iCCA studies and require direct validation in cHCC-CCA models.

Abbreviations: AFP, alpha-fetoprotein; ATG5/7, autophagy-related genes 5/7; CK7, cytokeratin 7; CK19, cytokeratin 19; DFS, disease-free survival; DRP1, dynamin-1-like protein; IHC, immunohistochemistry; LN, lymph node; MFN2, mitofusin 2; OS, overall survival; qPCR, quantitative polymerase chain reaction; TGF-β, transforming growth factor-beta; UPR, unfolded protein response.

## 4 Pathological diagnosis and differential diagnosis of cHCC-CCA

The current definition and diagnosis of cHCC-CCA are based on the histopathological identification of definitive hepatocellular and cholangiocytic differentiation (Tang et al., 2025). Within cHCC-CCA, the HCC and iCCA components may intermingle with either distinct or blurred boundaries, and in some cases, a discernible interface may be entirely absent (Komuta and Yeh, 2020). Due to the tumor’s high heterogeneity and the frequent sampling bias associated with biopsy specimens, distinguishing this entity from pure HCC or iCCA presents a significant diagnostic challenge (Ilyas et al., 2023; Wang H. et al., 2025).

In this context, immunohistochemistry (IHC) plays a crucial ancillary role (Ma L. et al., 2025) (Table 2). Typically, markers such as epithelial cell adhesion molecule (EpCAM), MOC31, epithelial membrane antigen (EMA), and cytokeratins 7 and 19 (CK7, CK19) are expressed in iCCA areas. In contrast, positivity for HepPar-1, Arginase-1, Alpha-fetoprotein (AFP), and CD10 suggests HCC differentiation (Yao et al., 2017). Notably, the transitional zones between the two components may co-express both biliary (CK7, CK19) and hepatocytic (HepPar-1, Arginase-1) markers. The diagnostic complexity is further compounded by the fact that some HCCs can atypically express biliary markers and exhibit fibrous stroma (Ilyas et al., 2023; Wang H. et al., 2025; Ma L. et al., 2025; Yao et al., 2017). However, the interpretation of these immunohistochemical markers is not without pitfalls (Ye et al., 2024). For example, a notable diagnostic challenge, particularly emphasized by recent literature, is the “epithelial EpCAM trap.” EpCAM is frequently used as a “stemness” marker, but its interpretation requires caution. EpCAM is positive in >90% of iCCA areas within cHCC-CCA, but only in 10%–20% of HCC areas. In pure HCC, EpCAM positivity occurs in approximately 35% of cases (Ye et al., 2024). This overlapping expression pattern creates a diagnostic dilemma, as a positive EpCAM stain in a tumor with ambiguous morphology might lead to an over-interpretation and an incorrect diagnosis. Given these diagnostic challenges, there is a growing need for adjunctive biomarkers that can provide prognostic information or help clarify tumor biology (Maharjan et al., 2022). A prospective study of 40 resected cHCC-CCA patients demonstrated that the autophagy markers LC3, Beclin1, and p62 are frequently overexpressed in tumor tissues, with positivity rates of 82.5%, 62.5%, and 76.5%, respectively (Maharjan et al., 2022). Notably, high intratumoral LC3 expression correlated with favorable clinicopathological features, such as reduced vascular invasion and lymph node metastasis, and emerged as an independent predictor of prolonged overall and disease-free survival after resection (Maharjan et al., 2022). Although not currently incorporated into the diagnostic criteria for cHCC-CCA, autophagy-related proteins have emerged as promising candidates.

**TABLE 2:** Classic immunohistochemical markers of cHCC-CCA(3-5, 13, 67).

Lineage	Recommended markers	Staining pattern	Caveats
Hepatocellular	HepPar-1, Arginase-1, AFP, CD10 (canalicular pattern)	Cytoplasmic (HepPar-1, Arginase-1, AFP); canalicular (CD10)	May be lost in poorly differentiated areas
Biliary	CK7, CK19, EMA, MOC31	Cytoplasmic/membranous	Can be aberrantly expressed in HCC (especially CK19^+^ HCC)
Progenitor/stem cell	EpCAM, CK19, CD56	Membranous/cytoplasmic	May lose markers during epithelial-mesenchymal transition

Immunohistochemical interpretation should consider tumor heterogeneity and potential aberrant expression; a panel of multiple markers is recommended for accurate diagnosis.

Abbreviations: AFP, alpha-fetoprotein; CK7, cytokeratin 7; CK19, cytokeratin 19; EMA, epithelial membrane antigen; EpCAM, epithelial cell adhesion molecule; GPC3, glypican-3; HepPar-1, hepatocyte paraffin antigen 1; MOC31, monoclonal antibody against epithelial glycoprotein; NCAM, neural cell adhesion molecule (CD56).

During the pathological diagnosis of cHCC-CCA, it is essential to differentiate it from three tumor types with overlapping morphological and immunophenotypic features, which differ fundamentally in biological behavior, prognosis, and treatment strategy (Komuta, 2022).

Although CK19-positive hepatocellular carcinoma (CK19+ HCC) does not form glandular structures, its expression of biliary markers such as CK19 and CK7 can lead to confusion with cHCC-CCA (Komuta, 2022; Zhuo et al., 2020). Despite the phenotypic overlap, CK19+ HCC lacks true bidirectional differentiation, which is a key diagnostic discriminator. Its overall survival (OS) typically falls between that of CK19- HCC and classical iCCA(70).

According to the WHO fifth edition classification, Cholangiolocellular carcinoma (CLC) is now reclassified as a small duct-type iCCA(71). It is architecturally characterized by ductular reactions within a prominent fibrous stroma and lacks an HCC component (Makino et al., 2024). Molecular evidence also supports its biliary origin. Therefore, in the complete absence of hepatocellular differentiation, a diagnosis of CLC, not cHCC-CCA, should be rendered (Steiner and Higginson, 1959).

Intermediate cell carcinoma, recognized as a subtype of cHCC-CCA. Its unique feature is that the tumor is composed entirely of uniform “intermediate” cells expressing both hepatocellular and cholangiocytic markers, without distinct classical HCC or iCCA areas (Beaufrere et al., 2021). These monomorphic cells exhibit a biphenotypic expression profile, and their invasive behavior can manifest features of both HCC and iCCA. This subtype is often found in the context of chronic liver disease (Beaufrere et al., 2021).

**FIGURE 3:**
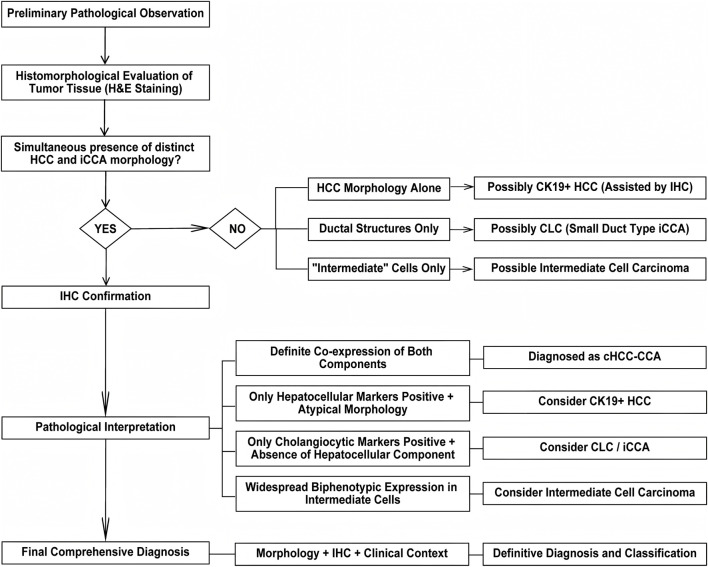
Diagnostic algorithm for cHCC-CCA based on histomorphology and immunohistochemistry. This flowchart outlines a stepwise diagnostic approach for suspected cHCC-CCA. Initial evaluation begins with histomorphological assessment using H&E staining to identify the presence of both HCC and iCCA components. If both components are clearly present, IHC is performed to confirm co-expression of lineage-specific markers. If only hepatocellular markers are positive in the context of atypical morphology, differential diagnoses such as CK19-positive HCC or CLC/iCCA should be considered. In cases where widespread biphenotypic expression is observed in intermediate cells without distinct classical areas, intermediate cell carcinoma is suggested. The final diagnosis integrates morphology, IHC results, and clinical context to achieve definitive classification. H&E, Hematoxylin and Eosin; HCC, Hepatocellular Carcinoma; iCCA, Intrahepatic Cholangiocarcinoma; cHCC-CCA, Combined Hepatocellular-Cholangiocarcinoma; IHC, Immunohistochemistry; CK19, Cytokeratin 19; CLC, Cholangiolocellular Carcinoma.

In summary, the pathological diagnosis of cHCC-CCA must rely on a comprehensive assessment integrating histological morphology with IHC markers. Given the significant tumor heterogeneity, particular vigilance is required to avoid misdiagnosis due to CK19+ HCC, CLC, and intermediate cell carcinoma (Steiner and Higginson, 1959; Wang et al., 2022). The judicious selection and interpretation of immunohistochemical markers, in conjunction with characteristic architectural features, are essential for accurate subtyping (Beaufrere et al., 2021; Sciarra et al., 2020) (Figure 3).

## 5 Systemic therapy for combined hepatocellular-cholangiocarcinoma

Due to its low incidence and high heterogeneity, there is currently no globally accepted standard systemic treatment regimen for cHCC-CCA, and clinical practice often references treatment strategies for HCC or CCA (73, 75). In recent years, several retrospective studies have explored the efficacy of different systemic therapeutic approaches in cHCC-CCA, providing limited evidence-based guidance for clinical decision-making (Table 3).

**TABLE 3:** Summary of major retrospective studies on systemic therapy for cHCC-CCA.

Study	Year	Sample size	Treatment regimen	ORR (%)	mPFS (months)	mOS (months)
(Tanabe et al., 2025)	2024	21	Lenvatinib	42.9	6.1	14.9
​	​	​	Atezolizumab + bevacizumab	14.3	7.9	Not reached
(Kim et al., 2021)	2021	99	Sorafenib	9.7	4.2	10.7
​	​	​	Cytotoxic chemotherapy	21.6	2.9	10.6
(Trikalinos et al., 2018)	2018	68	Gemcitabine + platinum	24.3	8.0	11.5
​	​	​	Gemcitabine ± fluorouracil	15.4	6.6	11.7
​	​	​	Sorafenib	0	4.8	9.6
(Salimon et al., 2018)	2018	30	Gemcitabine + platinum	28.6	9.0	16.2
(Kobayashi et al., 2018)	2018	36	Gemcitabine + cisplatin	5.6	3.8	11.9
​	​	​	Fluorouracil + cisplatin	—	3.0	10.2
​	​	​	Sorafenib	0	1.6	3.5
(Rogers et al., 2017)	2017	7	Gemcitabine + platinum ± bevacizumab	—	3.4	8.3

Regarding HCC-oriented treatment regimens, Tanabe et al. (2025) analyzed 21 patients receiving first-line therapy for cHCC-CCA and reported that lenvatinib achieved an objective response rate and disease control rate of 42.9% and 92.9%, respectively, with a median overall survival of 14.9 months. In comparison, atezolizumab plus bevacizumab demonstrated an overall response rate (ORR) of 14.3%, a disease control rate (DCR) of 100%, and median OS was not reached. These findings suggest that novel targeted and immunotherapeutic combinations developed for HCC may also hold potential value in cHCC-CCA. However, a larger study by Kim et al. (2021) encompassing 99 patients revealed no significant differences in ORR, progression-free survival, or overall survival (OS) between sorafenib and cytotoxic chemotherapy (such as platinum-based regimens), with ORRs of 9.7% and 21.6%, and median OS of 10.7 months and 10.6 months, respectively. This indicates that the advantage of conventional HCC-targeted therapy in this disease entity remains uncertain.

In terms of CCA-oriented treatment approaches, multiple studies support the efficacy of platinum-based chemotherapy. Salimon et al. (2018) analyzed 30 patients receiving gemcitabine plus platinum-based chemotherapy, reporting an ORR of 28.6%, and median progression-free survival (PFS) and OS of 9.0 months and 16.2 months, respectively. Kobayashi et al. (2018) analyzed 36 patients and similarly demonstrated that platinum-based regimens (such as gemcitabine plus cisplatin or fluorouracil plus cisplatin) conferred superior survival benefits compared to sorafenib monotherapy, with median OS of 10.2 months and 3.5 months, respectively. Trikalinos et al. (2018) further confirmed in 68 patients receiving systemic therapy that those treated with gemcitabine plus platinum achieved significantly better DCR compared to the gemcitabine plus fluorouracil group (78.4% vs. 38.5%, P = 0.008), with median PFS of 8.0 months and 6.6 months, respectively. In contrast, patients receiving sorafenib monotherapy had a DCR of only 20% and median PFS of 4.8 months, suggesting that platinum-based chemotherapy offers superior disease control in cHCC-CCA(80). Rogers et al. (2017) also noted favorable disease control in cHCC-CCA patients receiving gemcitabine plus platinum-based therapy, further supporting the clinical value of this regimen.

Collectively, the available evidence indicates that platinum-based chemotherapy (particularly gemcitabine combined with cisplatin or oxaliplatin) demonstrates relatively consistent efficacy advantages in cHCC-CCA, with DCR exceeding 70% and median OS ranging from 11 to 16 months. In contrast, conventional HCC-targeted agents such as sorafenib show limited efficacy, with most studies reporting low DCR and short PFS. Notably, novel targeted agents (such as lenvatinib) and immunotherapeutic combinations (such as atezolizumab plus bevacizumab) have shown promising potential in recent studies, despite limited case numbers. The encouraging ORR and OS data suggest that future treatment strategies may increasingly incline toward HCC-oriented approaches.

However, current studies have significant limitations: all are retrospective in design with relatively small sample sizes, inclusion criteria vary considerably, treatment selection is subject to bias, and most studies fail to perform stratified analyses based on the latest WHO pathological classification. Additionally, substantial differences in baseline patient characteristics (such as cirrhosis proportion, HBV/HCV infection rates, and prior treatment history) across studies may affect the comparability of results. Therefore, although existing evidence tends to support platinum-based chemotherapy as the first-line treatment option for cHCC-CCA, definitive recommendations cannot yet be established. Future prospective, multicenter studies incorporating molecular subtyping and biomarker analysis are urgently needed to explore more precise therapeutic strategies.

## 6 Discussion and future perspectives

Given the rarity and heterogeneity of cHCC-CCA, this review adopts a narrative approach to integrate current knowledge on its pathogenesis, molecular features, and therapeutic strategies, aiming to provide a comprehensive reference for clinicians and researchers. While this approach allows for a broad synthesis of the literature, it is not without limitations. The lack of a systematic search strategy and quantitative synthesis means that publication bias and study quality were not formally assessed. Nevertheless, by integrating findings across disciplines from cellular organelle dynamics to clinical management, this review offers a holistic perspective on a tumor entity that remains poorly understood.

A further limitation of this review, mirroring that of the field itself, is the paucity of direct mechanistic data in cHCC-CCA. Much of our understanding of organelle dysfunction is extrapolated from studies in HCC and iCCA. However, this limitation also presents an opportunity. By framing these extrapolations as hypothesis-generating, we hope to stimulate direct investigation into the organelle-level mechanisms that drive cHCC-CCA pathogenesis.

cHCC-CCA is a malignant neoplasm characterized by striking heterogeneity in its cellular origin, histological architecture, molecular phenotype, and clinical behavior (Komuta and Yeh, 2020; Beaufrere et al., 2021; Gentile et al., 2020). Its diagnosis and management have long suffered from a lack of standardized diagnostic criteria and consensus on management (Beaufrere et al., 2021). Although curative-intent surgery remains the most promising current approach, the high recurrence rate underscores the urgent clinical need to develop more effective adjuvant and systemic treatment strategies (Acalovschi, 2023). Available evidence supports the superior efficacy of iCCA-oriented chemotherapy, primarily based on gemcitabine-platinum regimens, in the majority of patients (Dageforde et al., 2021). Meanwhile, immunotherapy is emerging as a potential breakthrough, gradually entering the therapeutic landscape for cHCC-CCA (Unome et al., 2024). Encouragingly, several studies specifically targeting cHCC-CCA are now registered on the International Clinical Trials Registry Platform (ICTRP), reflecting significantly increased academic focus on this disease (Ye et al., 2024). However, due to its low incidence and diagnostic complexity, most existing research remains retrospective or based on small sample sizes, leaving a scarcity of high-quality evidence to guide clinical practice (Jang et al., 2023; Pomej et al., 2023).

Future research should prioritize several complementary directions to advance our understanding of organelle dysfunction in cHCC-CCA. First, direct organelle profiling in patient samples and cell lines using advanced techniques will be essential to establish whether the abnormalities observed are representative of the broader cHCC-CCA population, such as electron microscopy for ultrastructural analysis, organelle proteomics to characterize protein composition, and metabolomics to assess metabolic fluxes. Second, functional studies are urgently needed to test the specific roles of mitochondria, ER, and lysosomes in maintaining the biphenotypic state, these could employ pharmacological inhibitors or genetic perturbations in patient-derived models to determine whether disrupting individual organelle systems selectively impairs one lineage component. Third, therapeutic targeting of organelle pathways, including autophagy inhibitors, mitochondrial dynamics modulators, or ER stress inducers, should be evaluated in preclinical cHCC-CCA models, with particular attention to whether treatment efficacy correlates with the predominant lineage component. Finally, and perhaps most importantly for clinical translation, future studies must integrate organelle biology with clinical features, systematically exploring whether organelle phenotypes can predict treatment response or prognosis, thereby enabling precision medicine approaches that match organelle-targeting agents to tumor biology. Only by establishing a comprehensive research continuum, from elucidating molecular mechanisms to evaluating novel therapies, can we truly overcome the diagnostic and therapeutic challenges posed by cHCC-CCA and ultimately improve patient outcomes.

## Glossary

ADSL: Adenylosuccinate lyase
AFP: Alpha-fetoprotein
AKT: Protein Kinase B
ATG5/7: Autophagy-related genes 5/7
Beclin1: Bcl-2 interacting coiled-coil protein one
CCA: Cholangiocarcinoma
cHCC-CCA: Combined hepatocellular-cholangiocarcinoma
CK7: Cytokeratin 7
CK19: Cytokeratin 19
CLC: Cholangiolocellular carcinoma
DCR: Disease control rate
DFS: Disease-free survival
DRP1: Dynamin-1-like protein
EMA: Epithelial membrane antigen
EpCAM: Epithelial cell adhesion molecule
ER: Endoplasmic reticulum
ERAD: ER-associated degradation
EV: Extracellular vesicle
Foxl1: Forkhead box protein L1
HBV: Hepatitis B virus
HCC: Hepatocellular carcinoma
HCV: Hepatitis C virus
HPC: Hepatic progenitor cell
HSP90: Heat shock protein 90
ICTRP: International Clinical Trials Registry Platform
IDH: Isocitrate dehydrogenase
IHC: Immunohistochemistry
iCCA: Intrahepatic cholangiocarcinoma
INF2: Inverted formin 2
KDM8: Lysine demethylase 8
KRAS: Kirsten rat sarcoma viral oncogene homolog
LC3: Microtubule-associated protein 1 light chain 3
LD: Lipid droplet
LN: Lymph node
MAPK: Mitogen-activated protein kinase
Mdivi-1: Mitochondrial division inhibitor-1
MDV: Mitochondria-derived vesicle
MFN2: Mitofusin 2
mTOR: Mammalian target of rapamycin
NF-κB: Nuclear factor kappa B
N-RAS: Neuroblastoma RAS viral oncogene homolog
ORR: Objective response rate
OS: Overall survival
PCYT2: Phosphate cytidylyltransferase 2
PERK: Protein kinase R-like endoplasmic reticulum kinase
PFS: Progression-free survival
PI3K: Phosphatidylinositol 3-kinase
qPCR: Quantitative polymerase chain reaction
RER: Rough endoplasmic reticulum
ROS: Reactive oxygen species
SEL1L-HRD1: Suppressor of Lin-12-like protein 1 -HMG-CoA reductase degradation 1 homolog
SLC25A39: Solute carrier family 25 member 39
TERT: Telomerase reverse transcriptase
TGF-β: Transforming growth factor-beta
TP53: Tumor protein 53
UPR: Unfolded protein response
WHO: World Health Organization
Wnt: Wingless-related integration site
